# Glucose-regulated protein 78 and heparanase expression in oral squamous cell carcinoma: correlations and prognostic significance

**DOI:** 10.1186/1477-7819-12-121

**Published:** 2014-04-25

**Authors:** Fei Xia, Jin Cheng Xu, Pei Zhang, Yuan Yuan Zhang, Qian Wen Zhang, Zhen Hua Chao, Fang Wang

**Affiliations:** 1 Department of Stomatology, The First Affiliated Hospital of Bengbu Medical College, No 287, Chang-Huai Road, Bengbu 233000, Anhui, People’s Republic of China; 2 Department of Pharmacy, Bengbu Medical College, No 2600, East Sea Road, Bengbu 233000, Anhui, People’s Republic of China

**Keywords:** Glucose-related proteins 78, Heparanase, Immunohistochemistry, Oral squamous cell carcinoma, Prognosis

## Abstract

**Background:**

The aim of the present study was to investigate the expression of glucose-related protein 78 (GRP78) and heparanase (HPA) in oral squamous cell carcinoma (OSCC) and their relationship with clinicopathological parameters and potential implications for survival.

**Methods:**

A total of 46 patients with OSCC and 10 normal individuals were recruited for the study. GRP78 and HPA expression were determined in the lesion tissues using immunohistochemical analysis. The correlation between GRP78 and HPA was assessed using the Spearman correlation analysis. The associations of GRP78 and HPA with clinicopathological characteristics and survival were examined using the x^2^-test, Kaplan–Meier, or Cox regression.

**Results:**

Patients with OSCC showed a statistically significant higher prevalence of GRP78 and HPA expression than normal oral tissues. GRP78 and HPA expression was positively correlated with size, TNM stage, histological grade, lymphatic metastasis, and distant metastasis in OSCC patients. GRP78 expression was also positively correlated with HPA expression. Positive GRP78 and HPA expression was inversely correlated with survival in OSCC patients.

**Conclusions:**

HPA expression was found to be positively correlated with GRP78 expression. GRP78 and HPA are biomarkers that may have the potential to guide the treatment of oral cancer patients.

## Background

OSCC is the sixth most common malignancy in the world and has a worldwide incidence of over 263,900 new cases annually [1]. Despite numerous advances in diagnosis and treatment in the last half a century, such as surgery, chemotherapy, radiation, and other combinational therapies, mortality rates have remained unchanged. The five-year survival rate has remained at between 40 and 50% [2]. Currently, the treatment of choice for OSCC is considered by clinicopathological parameters. However, tumor outcome can differ even among patients whose tumors have the same clinicopathological parameters and who undergo the same treatments. This shows that individualized patient management is needed [3]. Tumor progression is a multifactorial, multi-step process, resulting in a heterogeneous group of cancers. Better understanding of the characteristics of tumors may ultimately help us provide patients with more individualized treatment. Recently, molecular-targeted therapy has drawn attention. This focuses on tumor cell specificity and the expression of molecules and proteins and their possible use in targeted therapy [4]. For molecular-targeted therapy to be effective, new factors for OSCC must be found.

GRP78 is an important member of the heat shock protein family 70 (HSPs70). It is localized to the endoplasmic reticulum (ER). GRP78 has many functions, but it plays a particularly essential role in protein biosynthesis. As a chaperone protein, it takes part in assembling, folding, and transporting newly synthesized proteins [5]. GRP78 also serves as a sensor for ER stress (ERS) under many ordinary physiological and pathological conditions, such as in patients with genetic mutations affecting secretory protein or chemical substances. These mutations can cause unfolded or misfolded proteins to accumulate in the lumen of the ER, resulting in ERS [6,7]. In order to sustain cytosolic ER homeostasis, cells activate a set of tightly controlled regulatory programs called the unfolded protein response (UPR) [6,7]. Some recent reports have demonstrated that regulation and expression of GRP78 are associated with resistance to apoptosis, and that overexpression of GRP78 may be involved in tumor progression in some forms of carcinoma [8,9]. However, tumor treatment failure is mainly attributable to metastasis. HPA is an endo-β-D-glucuronidase that specifically cleaves the side chains of heparan sulfate proteoglycans (HSPGs) [10]. For tumor cells to accomplish invasion and metastasis, they must first break through the extracellular matrix (ECM) and basement membrane (BM), which are mainly made up of HSPGs. Previous studies have shown that expression of HPA, which is the only enzyme capable of degrading HSPGs, is correlated to the degree of tumor invasion and metastasis in some malignancies [11,12]. Although the roles of GRP78 and HPA in the progression and prognosis of various human tumor tissues are widely recognized, there is a lack of reports on OSCC. Previous studies have shown that GRP78 is a marker of ERS, and that HPA can regulate the invasion and metastasis of tumor cells. When the tumor microenvironment becomes hypoxic, acidic, glucose-deprived, or is subjected to radiation treatment, tumor cells can invade, metastasize, and induce ERS, triggering UPR in tumor cells. The relationships among these factors have yet to be explained. With this in mind, immunohistochemistry was used to assess GRP78 and HPA expression in paraffin-embedded primary and metastatic tumor sections from patients with OSCC. The purpose of the present study was to investigate the correlations among GRP78, HPA, the clinicopathological parameters of OSCC, and prognostic significance. The results may positively influence the guiding of treatment for oral cancer patients.

## Methods

### Patients

In this retrospective study, 10 normal individuals with precancerous lesions and 46 patients with OSCC were recruited from the Department of Stomatology of the First Affiliated Hospital of Bengbu Medical College, (People’s Republic of China) between 2006 and 2008, and for whom tissue specimens from the local pathology repository and clinical follow up data were available. None of the patients had received irradiation or chemotherapy prior to surgery. All patients gave informed consent for participation and were interviewed uniformly before surgery by a well-trained interviewer. The questionnaire used in the interview addressed details regarding general demography, current and past cigarette smoking status, and alcohol consumption. Patients who had ever smoked cigarettes or drunk alcohol on a regular basis (at least once a week for one year) were classified as tobacco and alcohol users, respectively. For each patient, tumor stage and disease grade were classified by the pathologist according to guidelines published in the seventh edition of the TNM classification of the International Union against Cancer (UICC). The study was conducted according to the regulations of the local ethics committee and the laws of China.

### Patient characteristics and clinicopathological features

Demographic data and tumor characteristics are summarized in Table 1. A total of 46 patients were recruited in this retrospective study: 9 female and 37 male. The age of the participants ranged from 5 to 75 years (median 60 years), and the median tumor size was 3.1 cm (1 to 5.2 cm). The mean follow up period was 66 months (8 to 82 months). Overall, 84.8% (39 out of 46) of the patients were cigarette smokers, and 78.3% (36 out of 46) were alcohol drinkers. The histological tumor grades were G_1_-G_2_ in 12 cases and G_3_-G_4_ in 34 cases. Tests were positive for lymphatic invasion in 19 cases. The tumor stages were T_1_-T_2_ in 31 cases and T_3_-T_4_ in 15 cases. Distant metastasis at presentation (M_1_) was observed in 11 cases. Pathologic stages were I-II in 23 cases and III-IV in 23 cases.

**Table 1:** Correlations of GRP78 and HPA expression in oral cancer with the clinicopathological parameters

**Variable**	**n**	**%**	**GRP78**		***P***	**HPA**		***P***
			**Positive**	**Negative**		**Positive**	**Negative**	
**Tissue**					**0.003**			**0.033**
Normal	10	17.9	0	10		0	10	
OSCC	46	82.1	27	19		19	27	
**Gender**					**0.358**			**0.555**
Male	37	80.4	20	17		14	23	
Female	9	19.6	7	2		5	4	
**Age**					**0.606**			**0.055**
≤60	27	58.7	15	12		8	19	
>60	19	41.3	12	7		11	8	
**Tumor size**					**0.001**			**0.036**
≤3.1	23	50.0	8	15		6	17	
>3.1	23	50.0	19	4		13	10	
**Tumor stage**					**0.001**			**0.000**
T_1_-T_2_	31	67.4	13	18		7	24	
T_3_-T_4_	15	32.6	14	1		12	3	
**Lymphatic metastasis**					**0.000**			**0.012**
N_0_	27	58.7	10	17		7	20	
N_1-3_	19	41.3	17	2		12	7	
**Distant metastasis**					**0.033**			**0.000**
M_0_	35	76.1	17	18		8	27	
M_1_	11	23.9	10	1		11	0	
**TNM stage**					**0.000**			**0.000**
I-II	23	50.0	6	17		3	20	
III-IV	23	50.0	21	2		16	7	
**Histologic grade**					**0.018**			**0.016**
G_1_-G_2_	12	26.1	11	1		9	3	
G_3_-G_4_	34	73.9	16	18		10	24	
**Cigarette smoking**					**0.246**			**0.180**
Yes	39	84.8	21	18		14	25	
No	7	15.2	6	1		5	2	
**Alcohol drinking**					**0.787**			**1.000**
Yes	36	78.3	22	14		15	21	
No	10	21.7	5	5		4	6	

*P* <0.05 was considered to be statistically significant.

### Reagents

Goat monoclonal antibodies against human GRP78 and HPA were purchased from Santa Cruz Biotechnology (Santa Cruz, California, United States) and Abcam Biotechnology (Hong Kong, China). Elivison™ Plus Immunohistochemical Kits and Diaminobenzidine (DAB) Chromogenic Kits were purchased from Fuzhou Maixin Bio-technology Co. (Fuzhou, China). Phosphate-buffered saline (PBS, pH = 7.4) and sodium citrate-hydrochloric acid buffer solution (pH = 6.0) reagents were prepared in the Department of Pharmacy, Bengbu Medical College, Bengbu, Anhui, China.

### Immunohistochemistry

The expression of GRP78 and HPA was determined using Elivision™ immunohistochemical methods and DAB coloration. Briefly, the paraffin-embedded tumor sections (5 μm) were deparaffinized and dehydrated with graded alcohol. After washing for 10 minutes in PBS, the sections were heated for 15 minutes at 95°C in a sodium citrate-hydrochloric acid buffer solution to repair antigens. The endogenous peroxidase activity was quenched by incubation of the sections in methanol containing 3% H_2_O_2_ for 10 minutes at room temperature. After several washes in PBS, the sections were incubated with anti-GRP78 antibody (sc-13968) or anti-HPA antibody (ab85543) overnight at 4°C in a humidified chamber. Instead of reacting with the primary antibodies, the sections were treated with a polymer enhancer (reagent A, DAB buffer) for 20 minutes at room temperature. After a thorough wash in PBS, the sections were treated with the anti-goat antibody (reagent B, DAB substrate) for 30 minutes at room temperature. After being washed in PBS, they were developed in freshly prepared DAB solution for 8 minutes and then counterstained with hematoxylin, dehydrated, air-dried, and mounted onto slides.

### Evaluation of slides

The slides were evaluated by two independent observers using a light microscope in a blinded fashion. Cases of disagreement were re-evaluated on a double-headed microscope until consensus was reached. Ten visual fields were randomly selected from each slide. A score of 0 to 3, corresponding to colorless, faint yellow, yellow-brown, and brown, was given to evaluate the intensity of staining. A score of 0 to 3 was used to evaluate the relative number of positive cells as follows: samples in which less than 10% of the cells were positive were given a score of 0; samples in which between 10 and 50% of the cells were positive were given a score of 1; samples in which between 50 and 75% of the cells were positive were given a score of 2; samples in which more than 75% of the cells were positive were given a score of 3. The modified histochemical score was obtained by multiplying the score for the intensity of staining by the score for the relative number of positive cells. Based on this final score, results ≤1 were considered negative and those >1 positive.

### Statistical analysis

Statistical analysis was performed using the SPSS statistical package (SPSS, Chicago, Illinois, United States). The associations between GRP78, HPA expression, and clinicopathological parameters were evaluated using the x^2^-test. The correlation between GRP78 and HPA was assessed by the Spearman correlation analysis. Survival curves were generated using the Kaplan-Meier method and compared using the log-rank test. The Cox proportional hazard model was used to assess independent factors for the significance of the prognosis. *P* <0.05 was considered to be statistically significant.

## Results

### GRP78 and HPA expression in oral squamous cell carcinoma and normal tissue

**Figure 1:**
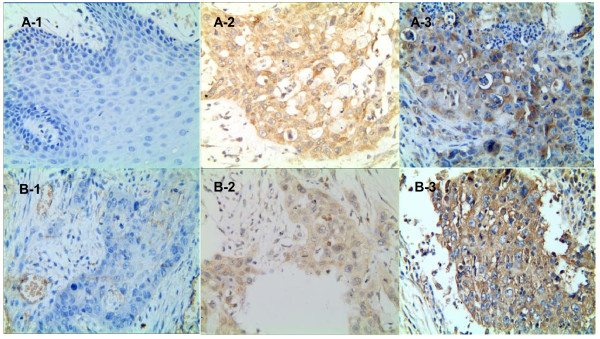
**Immunohistochemistry staining patterns of GRP78 and HPA expressions in normal tissues and OSCC. (A)** GRP78 expression in OSCC and normal tissues by immunostaining (×400). A-1: GRP78 in normal tissues: no expression; A-2: GRP78 in OSCC tissues: negative expression; A-3: GRP78 in OSCC tissues: positive expression. **(B)** HPA expression in OSCC and normal tissues by immunostaining (c × 400). B-1: HPA in normal tissues: no expression; B-2: HPA in OSCC tissues: negative expression; B-3: HPA in OSCC tissues: positive expression. GRP78, glucose-related protein 78; HPA, heparanase; OSCC, oral squamous cell carcinoma.

Clinical samples from 10 normal individuals and 46 OSCC patients were stained to confirm that GRP78 and HPA expression differed between these two groups. GRP78 was expressed in the cytoplasm in all OSCC samples (Figure 1). No GRP78 was expressed in any of the normal oral mucosa samples. In this way, there was a significant difference in the rate of expression of GRP78 between the OSCC group and the control group (*P* = 0.003). Similarly, HPA-positive expression signals were observed in 19 of 46 OSCC samples, but faint staining signals were observed in addition to the more commonly observed cytoplasmic staining. HPA was also found to localize in the nucleus and cytomembrane (Figure 1). There was a significant difference in the rate of expression of HPA between the OSCC group and the control group (*P* = 0.033).

### Correlations between GRP78 expression in oral squamous cell carcinoma and clinicopathological parameters

We observed a positive GRP78 expression in 27 of 46 OSCC samples (58.7%). The rate of GRP78 expression was found to be closely related to tumor size (>3.1 cm, *P* = 0.001), tumor stage (T_3_-T_4_, *P* = 0.001), lymphatic metastasis (N_1-3_, *P* = 0.000), distant metastasis (M_1_, *P* = 0.033), pathologic stage (III-IV, *P* = 0.000), and histological grade (G_3_-G_4_, *P* = 0.018). However, the rate of positive GRP78 expression showed no significant correlation with gender, age, cigarette smoking, or alcohol consumption (Table 1).

### Correlation between HPA expression in oral squamous cell carcinoma and clinicopathological parameters

HPA expression was observed in 19 of 46 OSCC samples (41.3%). The rate of HPA expression was found to be closely related to tumor size (>3.1; *P* = 0.036), tumor stage (T_3_ -T_4_; *P* = 0.000), lymphatic metastasis (N_1-3_; *P* = 0.012), distant metastasis (M_1_; *P* = 0.000), pathologic stage (III-IV; *P* = 0.000) and histological grade (G_3_-G_4_; *P* = 0.016). However, the rate of positive HPA expression showed no significant correlation with gender, age, cigarette smoking status, or alcohol consumption (Table 1).

### Correlations between GRP78 and HPA expression

There was a significant positive correlation between the expression of GRP78 and HPA (r = 0.435, *P* = 0.003) in OSCC patients (Table 2).

**Table 2:** Correlations of GRP78 and HPA expression in OSCC

**Variables**	**GRP78**		**r**	***P***
	**Positive**	**Negative**		
HPA			0.435	0.003
Positive	19	0		
Negative	8	19		

r,linear correlation; *P* <0.05 was considered to be statistically significant.

### Prognosis and multivariate analysis

**Figure 2:**
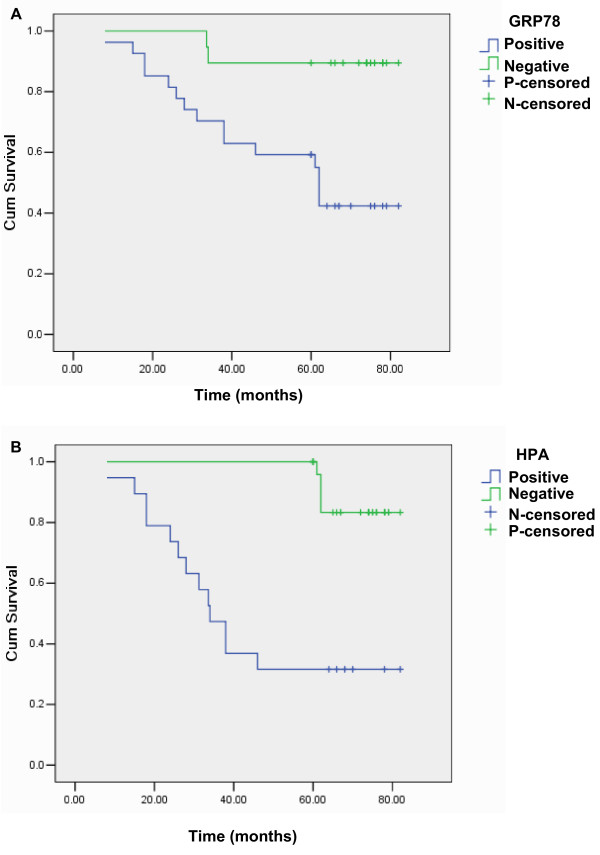
**Survival curves of the patients with OSCC. A**: correlation between positive or negative GRP78 expression and prognostic implication in OSCC patients. **B**: Correlation between HPA expression and prognostic implication in OSCC patients.

The survival rate of patients who were positive for GRP78 expression was significantly lower than that of patients who were negative for GRP78 expression (Log-rank Test, *P* = 0.002, Figure 2A). The survival rate of patients positive for HPA expression was significantly lower than that of patients negative for HPA expression (Log-rank Test, *P* = 0.000, Figure 2B). In addition, multivariate analysis showed age, distant metastasis, GRP78 expression, and HPA expression to be independent prognostic factors of OSCC (*P* <0.05) (Table 3).

**Table 3:** Cox regression analysis in overall survival of OSCC patients

**Risk factors**	**RR**	**95% CI**	***P***
**age**	**0.049**	**0. 007-0. 329**	**0. 002**
**Distant metastasis**	**7.566**	**1.243-46.049**	**0. 028**
**GRP78**	**0.023**	**0.002-0.239**	**0.002**
**HPA**	**0.08**	**0.001-0.110**	**0.000**

95% CI, 95% confidence interval; RR, relative risk.

## Discussion

Previous studies have shown that when the tumor microenvironment becomes hypoxic, acidic, glucose-deprived, or is subjected to radiation treatment, tumor cells can invade and undergo metastasis. The same conditions can also induce ERS. In the present study, correlations between HPA expression and OSCC invasion and metastasis were observed. Their possible implications in OSCC prognosis and survival were evaluated under ERS conditions. GRP78 is an ER-stationed chaperone protein that has been characterized on cell membranes and in the cytoplasm [5]. It is a central regulator of ER function because of its roles in protein folding and assembly, in the targeting of unfolded and misfolded proteins for degradation, ER Ca^2+^ binding, and control of the activation of transmembrane ERS sensors [6]. GRP78 is closely associated with ER stress-sensor proteins, such as inositol-requiring enzyme, protein kinase-like ER kinase, and activating transcription factor 6, but these sensors have not yet been reported to be correlated with the aggressiveness or progression of OSCC. For this reason, the present study focused on the GRP78 expression, which was believed to indicate the degree of ERS [13].

Previous studies have shown that a robust induction of GRP78 takes place in many malignancies. This has been described with respect to tumor progression and metastasis. For example, a high level of GRP78 has been shown to be more common in patients with high-grade breast cancer than in healthy individuals [14]. A similar pattern of expression was observed in lung cancer, prostate cancer, hepatocellular carcinoma, colon cancer, and gastric cancer [15-19]. The findings of the present study on excised OSCC were consistent with those of previous reports, and suggest that increased expression of GRP78 may be a feature of many malignant tumors found in humans. Some papers have shown that the regulation and expression of GRP78 are associated with shorter overall survival [20-22]. The present study demonstrated that positive GRP78 expression could also act as a predictor of shorter overall survival. Patients who were positive for GRP78 expression showed a higher risk of dying from OSCC than those with negative GRP78 expression. In this way, there is sufficient evidence to support the belief that GRP78 can serve as a marker of ERS, and that it may play an important role in the development and prognosis of OSCC.

The treatment of malignant tumors is a tough problem in the field of clinical oncology. Tumor metastasis is the cause of death of the majority of cancer patients. Previous studies have indicated that HPA expression is regulated by P53, NF-KB, and ERS to promote invasion and metastasis [23-25]. The present study focused on the relationship between GRP78 and HPA. Thirty out of a total of forty-six patients showed lymphatic metastasis or distant metastasis, and 90% were positive for GRP78 expression. It can be hypothesized that, under ERS conditions, HPA might be upregulated to help invasion and metastasis. HPA upregulation has been documented in many primary human tumors, such as bladder, gastric, colorectal, and renal cancer [26-29]. The activation of HPA enables tumor cells to break through the ECM and BM. HPA releases several types of cytokines, including vascular endothelial growth factor (VEGF) and basic fibroblast growth factor (bFGF). These cytokines are crucial to promoting cell movement, enhancing tumor cell invasion, and promoting tumor angiogenesis [30]. They are considered to be closely associated with invasion, metastasis, and prognosis in multiple types of malignant tumors. Data collected in the present study demonstrated that the expression of HPA in patients with either lymphatic metastasis or distant metastasis was higher than that in patients without metastasis, and there was a significant positive correlation between the expression of HPA and GRP78. In the present study, the HPA protein was found to be highly expressed in most advanced-stage OSCC tissues, and the proteins were mainly located within the cytoplasm and nucleus. These findings were consistent with those of a previously published study [31]. In a separate study Jiang *et al.* showed that knockdown of GRP78 can enhance the sensitivity of melanoma to chemotherapy drugs [32]. In this way, it can be hypothesized that removing the tumor protection provided by ERS can reduce the level of HPA and enhance the sensitivity of chemotherapy drugs. This may be one viable means of treating OSCC. However, the clinical significance of GRP78 and HPA in OSCC has not been fully explored thus far. A larger group of patients with long-term follow up is needed and more cellular experiments should be performed to confirm this conclusion.

## Conclusions

The current study is the first to explain the progression, metastasis, and prognosis of oral cancer progression from the perspective of ERS. HPA expression was found to be positively correlated with ERS. GRP78 and HPA are biomarkers that may have the potential to guide the treatment of oral cancer patients.

## Abbreviations

BFGF: basic fibroblast growth factor; BM: basement membrane; ECM: extracellular matrix; ER: endoplasmic reticulum; ER-stress: endoplasmic reticulum stress; GRP78: glucose-related protein 78; G: tumor grades; HPA: heparanase; HSPGs: heparan sulfate proteoglycans; HSPs70: heat shock protein family 70; M: distant metastasis; N: lymphatic metastasis; OSCC: oral squamous cell carcinoma; PBS: Phosphate-buffered saline; UPR: unfolded protein response; T: tumor stage; VEGF: vascular endothelial growth factor.

## Competing interests

The authors declare that they have no competing interests.

## Authors’ contributions

FX carried out experimental procedures and drafted manuscript. PZ, YYZ, QWZ, ZHC, and FW participated in the study design and were involved in the collection of clinical data and specimens. JCX designed the study and gave conceptual advice. All authors read and approved the final manuscript.
